# Serum neurofilament light chain in COVID-19 and the influence of renal function

**DOI:** 10.1186/s40001-023-01375-1

**Published:** 2023-09-28

**Authors:** Peter Körtvelyessy, Elena Diekämper, Klemens Ruprecht, Matthias Endres, Paula Stubbemann, Florian Kurth, Jan Adriaan Graw, Mario Menk, Jens Kuhle, Felix Wohlrab

**Affiliations:** 1 Department of Neurology, Charité – Universitätsmedizin Berlin, Freie Universität Berlin and Humboldt-Universität zu Berlin, Hindenburgdamm 30, 12200 Berlin, Germany; 2 German Center for Neurodegenerative Diseases (DZNE) in Magdeburg, 39120 Magdeburg, Germany; 3 German Center for Neurodegenerative Diseases (DZNE) in Berlin, 10117 Berlin, Germany; 4 Department of Pneumology, Charité – Universitätsmedizin Berlin, Freie Universität Berlin and Humboldt-Universität zu Berlin, 13533 Berlin, Germany; 5 Department of Anaesthesiology and Operative Intensive Care Medicine, Charité – Universitätsmedizin Berlin, Freie Universität Berlin and Humboldt-Universität zu Berlin, 13533 Berlin, Germany; 6 Department of Anesthesiology and Intensive Care Medicine, Universitätsklinikum Ulm, Ulm University, 89081 Ulm, Germany; 7 MS Center, Neurology and Research Center for Clinical Neuroimmunology and Neuroscience Basel, University Hospital and University Basel, Basel, Switzerland

**Keywords:** COVID-19, Neurofilament light chain, Creatinine, Biomarker

## Abstract

COVID-19 is associated with various neurological symptoms. Serum neurofilament light chain (sNfL) is a robust marker for neuroaxonal injury. Recent studies have shown that elevated levels of sNfL are associated with unfavorable outcome in COVID-19 patients. However, neuroaxonal injury is rare in COVID-19, and renal dysfunction and hypoxia, both of which are known in severe COVID-19, can also increase sNfL levels. Thus, the meaning and mechanisms of sNfL elevation in COVID-19 patients remain unclear. We evaluated sNfL levels in 48 patients with COVID-19 (mean age = 63 years) and correlated them to clinical outcome, the form of oxygen therapy, and creatinine. Levels of sNfL were age adjusted and compared with normal values and z-scores. COVID-19 patients treated with nasal cannula had normal sNfL levels (mean sNfL = 19.6 pg/ml) as well as patients with high-flow treatment (mean sNfL = 40.8 pg/ml). Serum NfL levels were statistically significantly higher in COVID-19 patients treated with mechanical ventilation on intensive care unit (ICU) (mean sNfL = 195.7 pg/ml, *p* < 0.01). There was a strong correlation between sNfL elevation and unfavorable outcome in COVID-19 patients (*p* < 0.01). However, serum creatinine levels correlated directly and similarly with sNfL elevation and with unfavorable outcome in COVID-19 patients (*p* < 0.01). Additionally, multivariate analysis for serum creatinine and sNfL showed that both variables are jointly associated with clinical outcomes. Our results identify renal dysfunction as an important possible confounder for sNfL elevation in COVID-19. Thus, serum creatinine and renal dysfunction should be strongly considered in studies evaluating sNfL as a biomarker in COVID-19.

## Background

Numerous neurological symptoms are associated with COVID-19, caused by SARS-CoV-2 infection, both in the acute phase of the disease and as long-term effects during post-acute sequelae of COVID-19. These include anosmia, dysgeusia, Guillain–Barré syndrome (GBS), stroke, encephalopathy, epilepsy, and neuropsychiatric symptoms [1–4]. Neurofilament light chain (NfL), as a constituent of the neuronal cytoskeleton, is a robust, well-known biomarker specific for neuroaxonal damage and has diagnostic and prognostic value in various neurological disorders such as amyotrophic lateral sclerosis, frontotemporal dementia, multiple sclerosis, or stroke [5–9]. Neurofilament light chain has been detected in serum (sNfL) and cerebrospinal fluid with commercially available assays. There are numerous studies showing the correlation between neurofilament light chain levels in serum or cerebrospinal fluid and neuroaxonal damage. Recent studies suggest sNfL as a potential neurological biomarker in COVID-19 addressing the putative neuroaxonal damage in COVID-19, which has not been proven up to today [10–12]. In one study hospitalized COVID-19 patients were found to have elevated sNfL levels regardless of neurological manifestations [13]. Another study demonstrated a correlation between sNfL levels and an unfavorable clinical outcome in COVID-19 patients [10]. Studies comparing sNfL in acute respiratory distress syndrome (ARDS) patients due to COVID-19 and bacterial pneumonia found elevated sNfL levels in both groups, suggesting a mechanism of sNfL elevation independent of SARS-CoV-2 in patients with ARDS [11, 12]. However, all of these studies did not consider factors influencing sNfL levels, such as renal dysfunction, hypoxia, or body mass index [14]. The exact pathomechanisms leading to COVID-19 causing neurological symptoms are still speculative, but the consequences of critical illness and hypoxia, hypercoagulopathy, para- and post-infectious inflammation, as well as the possible direct viral nerve infection may play a role [2, 15–19]. In a post-mortem study, myositis-like histopathology was found in COVID-19 patients, indirectly suggesting presumed retrograde neuroaxonal injury from muscle pathology, which is more prevalent in COVID-19 ICU patients than previously thought before [20]. Serum NfL levels remain highly elevated for months after brain injury, and higher sNfL levels have been shown to correlate with cardiovascular disease and peripheral neuropathies [14, 21–23]. In addition, sNfL levels are elevated in patients with subclinical ischemic events in the brain, as has been found in some COVID-19 patients [7]. Serum NfL levels are significantly elevated in patients after brain hypoxia and have better prognostic value than other serum markers including neuron-specific enolase, S100, and tau [24]. The latter results raise the question of whether hypoxia caused by COVID-19 pneumonia may affect sNfL levels.

On the other hand, it is known that non-neurological parameters such as renal dysfunction, diabetes mellitus, or body mass index can have an impact on sNfL levels [25, 26]. Especially, renal dysfunction is common in ICU patients and therefore a strong candidate of being responsible for increasing sNfL levels in ICU patients.

This study aimed to (i) examine sNfL in hospitalized COVID-19 patients, differentiated by oxygen treatment and clinical outcome, and (ii) test the hypothesis of whether sNfL level correlate with renal dysfunction or oxygen treatment in COVID-19 as a potential confounder of sNfL levels.

## Methods

### Study participants and recruitment

The participants of this study were prospectively recruited at COVID-19 patient care wards at the Charité Universitätsmedizin Berlin between March and October 2020. ICU patients with ARDS due to other than COVID-19 who were treated at Charité Universitätsmedizin Berlin were included retrospectively as a control group. All participants provided written informed consent, and all research was performed by following the relevant guidelines and regulations (PA_COVID study, approved by the ethical board of the Charité Universitätsmedizin Berlin, ethical approval number: EA2/066/20).

### COVID-19 diagnosis and patient categories

The diagnosis of SARS-CoV-2 infection was based on a polymerase chain reaction (PCR) test on nasopharyngeal swabs and clinical features of COVID-19 with fever and/or signs of respiratory insufficiency. A total of 48 patients with COVID-19 fulfilled all criteria of the study with creatinine and outcome data available. Neurological symptoms were reported in 8/48 patients comprising headache, dys-/anosmia or delirium. We divided COVID-19 patients into three different categories by the form of oxygen therapy: nasal cannula (NAC), non-invasive/high-flow therapy (NIV), and mechanical ventilation at the ICU.

### sNfL analysis and controls

The analysis of sNfL was done using the commercially available NF-light kit on the single molecule array (Simoa) HD-X Analyzer (Quanterix, Billerica, MA) as described before[11]. Serum samples were collected, processed, aliquoted and frozen for later use without freeze–thaw processes. Age-matched control normal values of sNfL were used as defined in Hvvid et al.[27]. Additionally, age-matched control values were compared to reference values from Benkert et al., yielding z-scores for each patient group [28]. Creatinine levels were retrospectively extracted from the electronic data sheet. Only those creatinine levels in samples taken at approximately (± 24 h) the same time as the serum samples for sNfL measurements were taken into account.

### Clinical severity assessment

We evaluated the grade of clinical severity in all patients using the standardized COVID-19 ordinal outcome scale (OOS) as proposed by the WHO ranging from 1 point (= no limitations of activities) to 8 points (= dead) (Table 1). The OOS was raised at two different time points. The clinical outcome examination at the first time point, defined as the first clinical outcome scale, was taken at -3 to 0 days before discharge, the second time point, defined as the second clinical outcome scale, was taken after discharge in a follow-up examination with the time point depending on the severity of the disease.

**Table 1:** Ordinal Outcome Scale

1	No limitations
2	Slight limitations in everyday life
3	On a normal ward without oxygen supply
4	On a normal ward with oxygen supply
5	On a ward with high-flow or non-invasive oxygen supply
6	Intensive care unit ventilated
7	Intensive care unit ventilated plus extra-corporal replacement therapy
8	Death

### Comparison with ARDS patients of other etiologies

ARDS patients of other etiologies than COVID-19 were included (*n* = 4) to compare with ARDS patients due to COVID-19. All ARDS patients fulfilled the criteria of the Berlin Definition for ARDS [29] and were admitted to the tertiary ARDS referral center of the Department of Anesthesiology and Intensive Care Medicine, between January 2013 and December 2018, a long time before the SARS-CoV-2 virus emerged [30].

### Statistical Analysis

Statistical analyses were performed with the software SPSS Statistics (IBM). Data of serum levels of NfL and creatinine were log transformed when using parametric tests. We used one-way ANOVA, *t*-test for independent samples, Pearson analysis, Spearman rho, and a multivariate analysis (ANCOVA). We considered *p* < 0.05 as statistically significant.

## Results

### Epidemiology

Overall, we included 48 patients (median age was 63 years, range between 21 and 90 years, 31 male and 17 female). 16 out of 48 (33.3%) patients were treated with oxygen therapy via NAC. 11 out of 48 (22.9%) patients received treatment with NIV and 21 out of 48 (43.8%) patients underwent mechanical ventilation therapy. Sampling was done at a mean of 8.4 (1–30) days after admittance in the NAC group, at a mean of 4.5 (1–17) days in the NIV group, and at a mean of 11.6 (4–18) days in the ICU group (Table 2). Additionally, we assessed whether there were any neurological symptoms described and found three cases of delirium and five cases of hyposmia in the cohort of 48 patients. None of the COVID-19 patients at the ICU had documented signs of a critical illness myopathy or neuropathy reported at the time of sampling. The four ARDS patients (median age 57 years) suffered from CIP/CIM at the time point of sample taking. Two of them died, and two remained on mechanical ventilation at short-term follow-up. Unfortunately, we did not have data on the body mass index (BMI) of our patients, as the BMI is also a known important sNfL confounder [14].

**Table 2:** Epidemiological data

	Normal ward (NAC)	High flow (NIV)	ICU	ARDS
*n* =	16	11	21	4
Sex (f:m)	9:7	0:11	4:17	1:3
Age (median)	52	65	71	57
Outcome at discharge	3.0	3.7	6	7.5
Patients died	0	1	9	2
Serum NfL (mean) [pg/ml]	18.8	44.0	195.7	487.8
Normal serum NfL (age adjusted, pg/ml)	20.5	29.9	30.9	20.9
Patients with normal serum NfL	10	8	2	0
Creatinine (mean) [mg/dl]	0.8	0.9	1.4	1.69
Time of sampling after admission	8.4 days	4.6 days	11.7 days	Not known
1st OOS (before discharge) mean	3.00	3.72	6.10	Not known
2nd OOS (after discharge) mean	1.00	2.33	3.5	Not known

### Differentially elevated sNfL levels depending on O_*2*_ treatment and clinical outcome

**Fig. 1:**
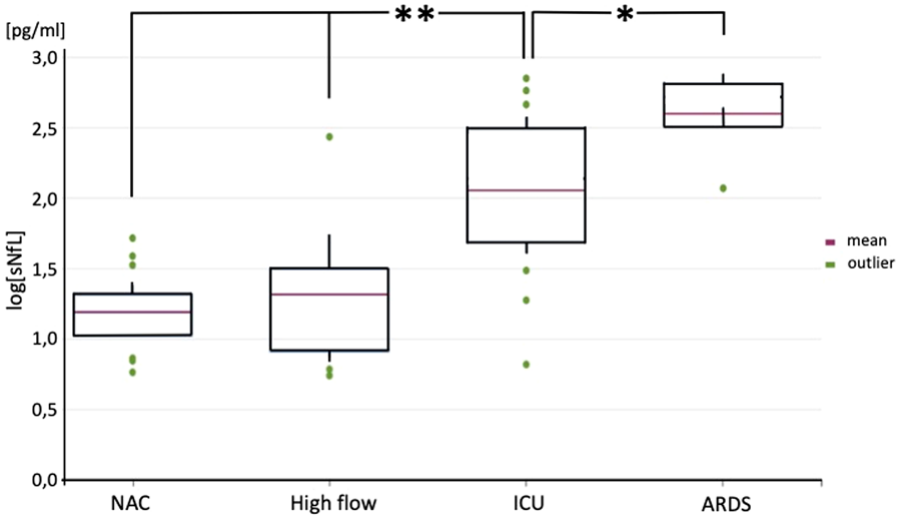
Boxplot visualization of ANOVA post hoc analysis: sNfL levels are differentially elevated in different treatment groups. Log[sNfL] (sNfL, log_10_-transformed) is elevated in mechanically ventilated ARDS-ICU patients (abbreviated as ICU) with COVID-19 and is even higher elevated in mechanically ventilated ARDS-ICU patients of other causes than COVID-19 (abbreviated as ARDS), compared to patients receiving treatment on a regular ward with either nasal cannula oxygen supply (abbreviated as NAC) or non-invasive/high-flow therapy (abbreviated as High flow) * *p*-value < 0.05; ** *p*-value < 0.01

First, we analyzed the sNfL values of COVID-19 patients, who were treated via NAC or NIV/high-flow therapy. No statistically significant differences in sNfL levels in the NAC and NIV groups compared to age-matched controls were found using a t-test for independent samples. In detail, we found sNfL values with a mean of 19.6 pg/ml (standard deviation (SD) = 13.4) for NAC versus the age-matched control at a mean of 27.6 pg/ml (SD = 14.5) resulting in a p-value of 0.612. In the NIV cohort sNfL was at a mean 40.8 pg/ml (SD = 74.6) compared to age-matched control mean of 27.6 pg/ml (SD = 14.5) resulting in a p-value of 0.55. Next, we compared all treatment groups including the ICU patient groups. An ANOVA post hoc analysis showed that sNfL levels were significantly elevated (p-value < 0.001) in both ICU patient groups treated by mechanical ventilation due to COVID-19 (mean = 195.7 pg/ml, SD = 193.0) and ARDS due to pneumonia of other cause than COVID-19 (mean = 487.7 pg/ml, SD = 242.5) also when compared to COVID-19 patients in treatment via NAC (mean = 19.6 pg/ml, SD = 13.4) or by NIV (mean = 40.8 pg/ml, SD = 74.6) (Fig. 1).

Additionally, we evaluated the z-scores for sNfL in all patient groups using the sNfL Reference App developed by Benkert et al.[28]. Here, we found normal z-scores for the NAC and NIV groups (NAC: mean = 1.43 (SD = 0.75), NIV: mean = 1.05 (SD = 1.56)) and strongly elevated z-scores for the ICU and ARDS groups (ICU: mean = 3.11 (SD = 0.73), ARDS mean = 3.83 (SD = 0.36)), which additionally confirms our first result using another method.

Next, we performed a correlation analysis between initial sNfL and clinical outcomes in COVID-19 patients. 9 of 21 patients treated at the ICU died (first and second OOS of 8 points) and 12 of 21 patients had a first mean OOS of 6.7 points and a second mean OOS of 6.0 points with a mean interval between both OOS of 75 days (Table1). COVID-19 patients treated with NIV had a first mean OOS of 3.7 points and a second mean OOS of 2.3 points 41 days later. Patients treated with NAC had a mean OOS with 3.0 points at the first assessment and a mean of 1.0 points at the second assessment 109 days later.

**Fig. 2:**
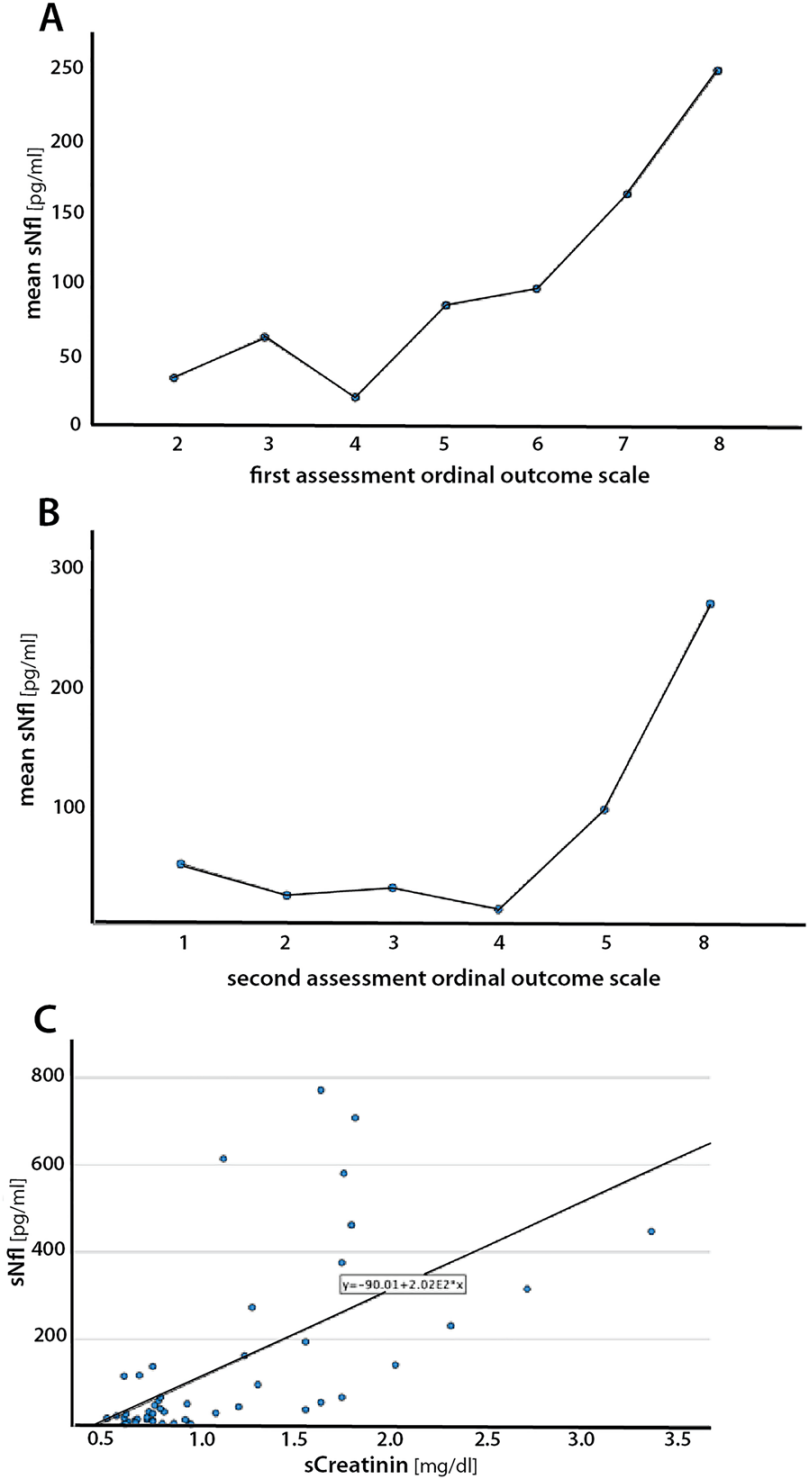
Spearman Rho Correlation Analysis: We categorized the COVID-19 patients into groups separated by the ordinal outcome scale (OOS). Serum NfL [pg/ml] correlates with an intermediate, unfavorable outcome in COVID-19 patients. **A** On the x-axis, the first OOS is represented with ordinal variables between 0 and 8. On the y-axis, the mean values of sNfL measured in pg/ml are represented. **B** On the x-axis, the second OOS is represented with ordinal variables between 0 and 8. On the y-axis, the mean values of sNfL measured in pg/ml are represented. **C** A Pearson correlation analysis showed that sNfL correlates significantly with creatinine in COVID-19 patients and ARDS patients. On the x-axis, creatinine values [mg/dl] and on the y-axis sNfL values are shown as measured in all 48 patients of this study

Spearman rho correlation analysis showed that the clinical outcome measured by the OOS is strongly correlated to initial sNfL levels with higher sNfL levels correlating with an unfavorable clinical outcome. The correlation coefficient for sNfL and the first clinical outcome scale was ρ = 0.484, *p*-value < 0.001 (Fig. 2a), and the correlation coefficient for sNfL and the second clinical outcome scale was ρ = 0.508, *p*-value < 0.001 (Fig. 2b).

Additionally, we examined whether sNfL levels correlate with the duration of ICU therapy by using a Pearson correlation analysis. There was no statistically significant correlation for sNfL and the duration of ICU therapy (ρ =− 0.203, *p*-value = 0.377).

### Creatinine levels correlate with sNfL and outcome

To test whether sNfL elevation was due to renal failure in COVID-19 patients with ARDS requiring mechanical ventilation due to COVID-19 or pneumonia of other cause than COVID-19, creatinine and sNfL in patients of all groups were examined. Pearson correlation analysis showed a strong positive correlation between creatinine and sNfL (correlation coefficient = 0.63, *p*-value < 0.001) with increased creatinine levels correlating with elevated sNfL values (Fig. 2c).

**Fig. 3:**
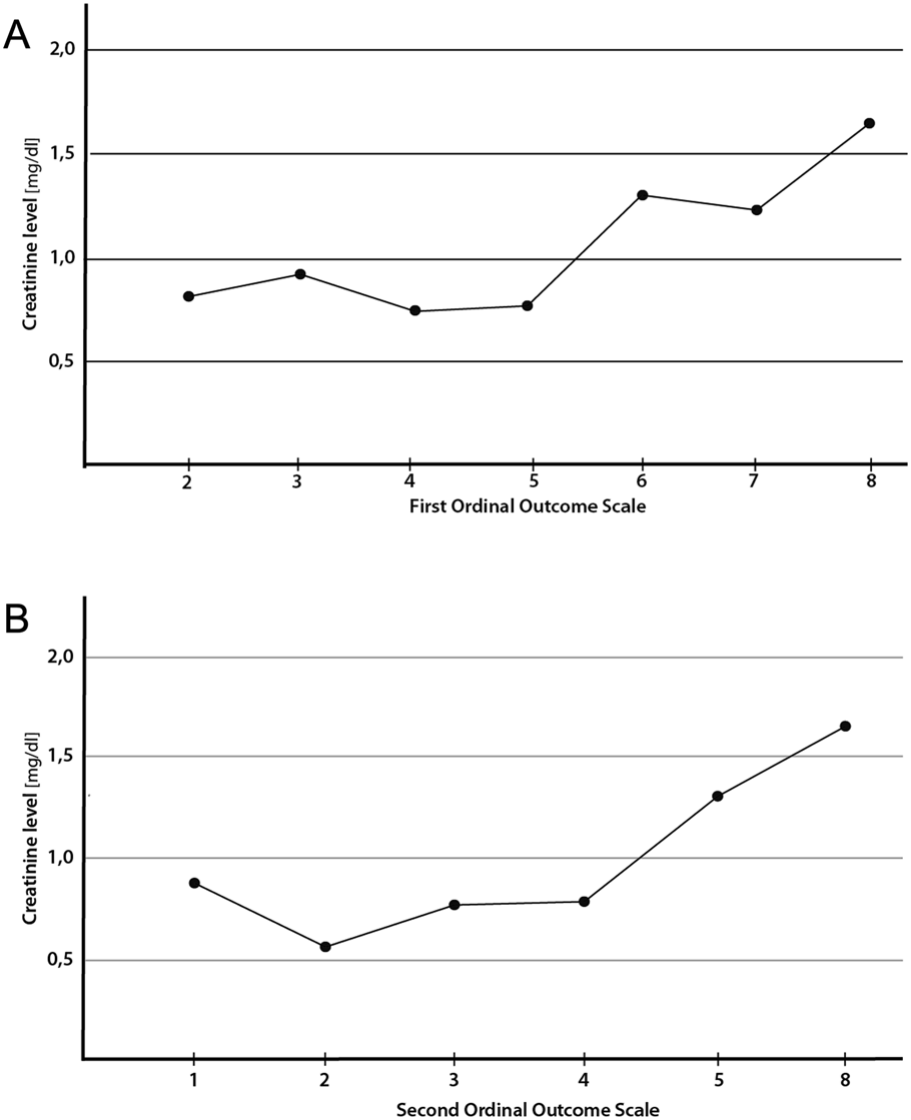
Line Plot: We categorized the COVID-19 patients into groups separated by the ordinal outcome scale (OOS). Creatinine correlates with an intermediate, unfavorable outcome in COVID-19 patients. **A** On the x-axis, the first OOS is represented with ordinal variables between 0 and 8. On the y-axis, the mean values of creatinine measured in mg/dl are represented. **B** On the x-axis, the second OOS is represented with ordinal variables between 0 and 8. On the y-axis, the mean values of creatinine measured in mg/dl are represented

We then investigated the correlation between creatinine and outcome as measured by the first OOS (Fig. 3a). We found a strong positive correlation (correlation coefficient = 0.56, *p*-value < 0.001) between creatinine and outcome at discharge as measured by the first OOS. Also, the correlation between creatinine and outcome as measured by the second OOS was significant with *p*-value < 0.001 and rho = 0.65 (Fig. 3b).

To further dissect the correlation of both, sNfL and creatinine, with clinical outcomes in COVID-19 patients, we performed a multivariate analysis for the clinical outcome as measured by the first OOS considered jointly on the variables creatinine and sNfL. There was a statistically significant effect on clinical outcome scale values when considered jointly on the variables creatinine and sNfL with a Wilks’ Lambda = 0.578, *F* = 1.99, and *p*-value = 0.036.

## Discussion

We analyzed sNfL and creatinine levels in three different COVID-19 patient groups, which were separated by the severity of respiratory illness as defined by the categorical intensity of respiratory therapy. Our data show that sNfL and creatinine correlate similarly with the outcome of COVID-19 patients. These results strongly suggest that both, creatinine and sNfL, can be used equally as a prognostic biomarker in COVID-19 and that sNfL elevation in COVID-19 is more likely due to renal dysfunction than to neuroaxonal damage during SARS-CoV-2 infection.

Patients treated with mechanical ventilation on ICU had significantly higher sNfL levels compared with the patients with NAC or NIV/high-flow therapy. In general, patients who are mechanically ventilated have a short phase of hypoxia before being intubated, with hypoxia potentially inducing the release of sNfL as described in patients with longer phases of hypoxia [24].

Another reason for elevated sNfL levels might be critical illness polyneuropathy (CIP) or critical illness myopathy (CIM) which was diagnosed in patients of our ARDS control group. However, our COVID-19 ICU patients had no reported symptoms indicative of CIP/CIM at the time of sampling. In addition, developing a CIP/CIM during a short period of time at the ICU with a mean of 11.6 days after admittance to the hospital and 10.6 days at the ICU is not very likely. Nevertheless, when comparing the mean sNfL in the COVID-19 ICU group (sNfL = 197.5 pg/ml) and the ARDS group (sNfL = 465.7 pg/ml), the comorbidity of CIP/CIM in the ARDS patients might be a contributing factor to additional sNfL elevation, as sNfL is known to be released following to damage of peripheral nerves [21–23, 31].

When examining the correlation of sNfL and creatinine in our cohort, a clear and highly significant correlation was seen. Previous analyses showed that sNfL is elevated in COVID-19 and correlates with disease severity [10, 13]. However, in these studies, renal dysfunction has not been analyzed. Our data suggest that sNfL elevation in COVID-19 patients is mainly due to a critical illness-associated renal dysfunction with subsequent impaired renal elimination of sNfL, rather than due to direct neuroaxonal damage [25].

This hypothesis is additionally supported by the result that there was no significant elevation of sNfL levels in COVID-19 patients in the non-critical ill patient groups (NAC and NIV groups) and the results of a multivariate analysis showing that sNfL and creatinine jointly contribute to poor clinical outcome and not sNfL alone.

Also, our data challenge the notion in recent publications that sNfL elevation is a specific observation in SARS-CoV-2-associated pneumonia [10]. In our study, we could show that sNfL elevations are even higher in a subgroup of severely ill pneumonia patients due to other pathogens than SARS-CoV-2, for example, due to Influenza A virus in one patient. This finding confirms the results of another recent publication in which sNfL has been shown to be elevated in critically ill patients with pneumonia due to SARS-CoV-2 as well as due to other bacterial pathogens with an even higher elevation of sNfL in patients with bacterial pneumonia [12]. It is unknown whether the same observations can be made in general in patients with ARDS due to other viral diseases since there are no studies covering this topic until today. This should be tested in upcoming additional studies.

Our study is mainly limited by the small sample size, due to our complex inclusion criteria, and therefore, we were not able to unravel a potential additional benefit of sNfL measurements next to creatinine in COVID-19 patients. Also, the numbers are too small to address gender differences in our cohort.

## Conclusion

In summary, our data suggest that it is not COVID-19 itself, but rather the severity of COVID-19 respiratory disease requiring ICU treatment and concomitant renal dysfunction that leads to elevated sNfL levels, and both, sNfL and creatinine, are associated with poor clinical prognosis. Other studies on the correlation of neurofilament light chain levels and COVID-19, and in general, studies with patients treated on ICU should consider this important link.

## Data Availability

All data are available by request to the corresponding author.

## Abbreviations

ARDS: Acute respiratory distress syndrome
BMI: Body mass index
COVID-19: Coronavirus disease 2019
ICU: Intensive care unit
NAC: Nasal cannula oxygen treatment
NfL: Neurofilament light chain
NIV: Non-invasive ventilation
SARS-CoV-2: Severe acute respiratory syndrome coronavirus type 2
SD: Standard deviation
sNfL: Serum neurofilament light chain
OOS: Ordinal outcome scale
PCR: Polymerase chain reaction
WHO: World Health Organization

## References

[1] Helms J, Kremer S, Merdji H, Clere-Jehl R, Schenck M, Kummerlen C (2020). Neurologic features in severe SARS-CoV-2 infection. N Engl J Med.

[2] Iadecola C, Anrather J, Kamel H (2020). Effects of COVID-19 on the nervous system. Cell.

[3] Gutiérrez-Ortiz C, Méndez A, Rodrigo-Rey S, San Pedro-Murillo E, Bermejo-Guerrero L, Gordo-Mañas R (2020). Miller Fisher syndrome and polyneuritis cranialis in COVID-19. Neurology.

[4] Paterson RW, Brown RL, Benjamin L, Nortley R, Wiethoff S, Bharucha T (2020). The emerging spectrum of COVID-19 neurology: clinical, radiological and laboratory findings. Brain.

[5] Feneberg E, Oeckl P, Steinacker P, Verde F, Barro C, Van Damme P (2018). Multicenter evaluation of neurofilaments in early symptom onset amyotrophic lateral sclerosis. Neurology.

[6] Kuhle J, Kropshofer H, Haering DA, Kundu U, Meinert R, Barro C (2019). Blood neurofilament light chain as a biomarker of MS disease activity and treatment response. Neurology.

[7] Tiedt S, Duering M, Barro C, Boeck AGJ, Bode FJ, Klein M (2018). Serum neurofilament light a biomarker of neuroaxonal injury after ischemic stroke. Neurology.

[8] Barro C, Benkert P, Disanto G, Tsagkas C, Amann M, Naegelin Y (2018). Serum neurofilament as a predictor of disease worsening and brain and spinal cord atrophy in multiple sclerosis. Brain.

[9] Körtvelyessy P, Heinze HJ, Prudlo J, Bittner D (2018). CSF biomarkers of neurodegeneration in progressive non-fluent aphasia and other forms of frontotemporal dementia: clues for pathomechanisms?. Front Neurol.

[10] Prudencio M, Erben Y, Marquez CP, Jansen-West KR, Franco-Mesa C, Heckman MG (2021). Serum neurofilament light protein correlates with unfavorable clinical outcomes in hospitalized patients with COVID-19. Sci Transl Med.

[11] Sutter R, Hert L, De Marchis GM, Twerenbold R, Kappos L, Naegelin Y (2021). Serum neurofilament light chain levels in the intensive care unit: comparison between severely Ill patients with and without coronavirus disease 2019. Ann Neurol.

[12] Chung H-Y, Neu C, Wickel J, Kuckertz SL, Coldewey SM (2021). Neurofilament light chain in patients with COVID-19 and bacterial pneumonia. Ann Neurol.

[13] Paterson RW, Benjamin LA, Mehta PR, Brown RL, Athauda D, Ashton NJ (2021). Serum and cerebrospinal fluid biomarker profiles in acute SARS-CoV-2-associated neurological syndromes. Brain Commun.

[14] Barro C, Chitnis T, Weiner HL (2020). Blood neurofilament light: a critical review of its application to neurologic disease. Ann Clin Transl Neurol.

[15] Jesuthasan A, Massey F, Manji H, Zandi MS, Wiethoff S (2021). Emerging potential mechanisms and predispositions to the neurological manifestations of COVID-19. J Neurol Sci.

[16] Johansson A, Mohamed MS, Moulin TC, Schiöth HB (2021). Neurological manifestations of COVID-19: a comprehensive literature review and discussion of mechanisms. J Neuroimmunol.

[17] Meinhardt J, Radke J, Dittmayer C, Franz J, Thomas C, Mothes R (2020). Olfactory transmucosal SARS-CoV-2 invasion as a port of central nervous system entry in individuals with COVID-19. Nat Neurosci.

[18] Matschke J, Lüthgehetmann M, Hagel C (2020). Neuropathology of patients with COVID-19 in Germany: a post-mortem case series. Lancet Neurol.

[19] Reinhold D, Farztdinov V, Yan Y, Meisel C, Sadlowski H, Kühn J (2022). The brain reacting to COVID-19: analysis of the cerebrospinal fluid and serum proteome transcriptome and inflammatory proteins. medRxiv.

[20] Aschman T, Schneider J, Greuel S, Meinhardt J, Streit S, Goebel H-H (2021). Association between SARS-CoV-2 infection and immune-mediated myopathy in patients who have died. JAMA Neurol.

[21] Huehnchen P, Schinke C, Bangemann N, Dordevic AD, Kern J, Maierhof SK (2022). Neurofilament proteins as a potential biomarker in chemotherapy-induced polyneuropathy. JCI Insight.

[22] Körtvelyessy P, Kuhle J, Düzel E, Vielhaber S, Schmidt C, Heinius A (2020). Ratio and index of neurofilament light chain indicate its origin in guillain-barré syndrome. Ann Clin Transl Neurol.

[23] Schinke C, Fernandez Vallone V, Ivanov A, Peng Y, Körtvelyessy P, Nolte L (2021). Modeling chemotherapy induced neurotoxicity with human induced pluripotent stem cell (iPSC) -derived sensory neurons. Neurobiol Dis.

[24] Moseby-Knappe M, Mattsson N, Nielsen N, Zetterberg H, Blennow K, Dankiewicz J (2019). Serum neurofilament light chain for prognosis of outcome after cardiac arrest. JAMA Neurol.

[25] Akamine S, Marutani N, Kanayama D, Gotoh S, Maruyama R, Yanagida K (2020). Renal function is associated with blood neurofilament light chain level in older adults. Sci Rep.

[26] Polymeris AA, Helfenstein F, Benkert P, Aeschbacher S, Leppert D, Coslovsky M (2022). Renal function and body mass index contribute to serum neurofilament light chain levels in elderly patients with atrial fibrillation. Front Neurosci.

[27] Hviid CVB, Knudsen CS, Parkner T (2020). Reference interval and preanalytical properties of serum neurofilament light chain in Scandinavian adults. Scand J Clin Lab Invest.

[28] Benkert P, Meier S, Schaedelin S, Manouchehrinia A, Yaldizli Ö, Maceski A (2022). Serum neurofilament light chain for individual prognostication of disease activity in people with multiple sclerosis: a retrospective modelling and validation study. Lancet Neurol.

[29] Marco Ranieri V, Rubenfeld GD, Taylor Thompson B, Ferguson ND, Caldwell E, Fan E, Fan E, Camporota LSA (2012). Acute respiratory distress syndrome: the Berlin definition. JAMA.

[30] Hunsicker O, Materne L, Bünger V, Krannich A, Balzer F, Spies C (2020). Lower versus higher hemoglobin threshold for transfusion in ARDS patients with and without ECMO. Crit Care.

[31] Maia LF, Maceski A, Conceição I, Obici L, Magãlhes R, Cortese A (2020). Plasma neurofilament light chain: an early biomarker for hereditary ATTR amyloid polyneuropathy. Amyloid.

